# Impact of radiation dose in postoperative radiotherapy after R1 resection for extrahepatic bile duct cancer: long term results from a single institution

**DOI:** 10.18632/oncotarget.17368

**Published:** 2017-04-21

**Authors:** Byoung Hyuck Kim, Eui Kyu Chie, Kyubo Kim, Jin-Young Jang, Sun Whe Kim, Do-Youn Oh, Yung-Jue Bang, Sung W. Ha

**Affiliations:** ^1^ Department of Radiation Oncology, Seoul National University College of Medicine, Seoul, Republic of Korea; ^2^ Department of Surgery, Seoul National University College of Medicine, Seoul, Republic of Korea; ^3^ Department of Internal Medicine, Seoul National University College of Medicine, Seoul, Republic of Korea; ^4^ Division of Biological Warfare Preparedness and Response, Armed Forces Medical Research Institute, Daejeon, Republic of Korea; ^5^ Department of Radiation Oncology, Ewha Womans University School of Medicine, Seoul, Republic of Korea

**Keywords:** extrahepatic bile duct cancer, R1 resection, postoperative radiotherapy, radiation dose

## Abstract

**Purpose:**

This study was conducted to evaluate the impact of radiation dose after margin involved resection in patients with extrahepatic bile duct cancer.

**Methods:**

Among the 251 patients who underwent curative resection followed by adjuvant chemoradiotherapy, 86 patients had either invasive carcinoma (*n* = 63) or carcinoma in situ (*n* = 23) at the resected margin. Among them, 54 patients received conventional radiation dose (40-50.4 Gy) and 32 patients received escalated radiation dose (54-56 Gy).

**Results:**

Escalated radiation dose was associated with improved locoregional control (5yr rate, 73.8% *vs*. 47.1%, *p* = 0.069), but not disease-free survival (5yr rate, 43.4% *vs*. 32.6%, *p* = 0.490) and overall survival (5yr rate, 40.6% *vs*. 29.6%, *p* = 0.348). In multivariate analysis for locoregional control, invasive carcinoma at the margin (HR 2.957, *p* = 0.032) and escalated radiation dose (HR 0.394, *p* = 0.047) were independent prognostic factors. No additional gastrointestinal toxicity was observed in escalated dose group.

**Conclusions:**

Delivery of radiation dose ≥ 54 Gy was well tolerated and associated with improved locoregional control, but not with overall survival after margin involved resection. Further validation study is warranted.

## INTRODUCTION

Tumor of the extrahepatic bile duct (EHBD) is a rare form of gastrointestinal tract cancer with poor prognosis [1, 2]. Although a radiologic evaluation on the extent of tumor of EHBD has improved over the years, it is still quite challenging to accurately assess required surgical extent and thus status of resection margins (RM) prior to actual resection procedure [3–5]. The presence of unexpected infiltrative disease along biliary tract and complex anatomic structures around the tumor make an R0 resection challenging. Thus, microscopic positive (R1) resection seems inevitable, especially in patients with high operative risk.

Despite the effort of aggressive surgery including portal vein resection and hepatopancreatoduodenectomy to achieve a higher R0 resection rate, the reported incidence of a R1 RM after intended curative resection has varied from 20% to as high as 60 % in the literature [6–10]. These wide-ranged incidences of R1 RM were explained by a variation between the definition of R1 RM or the operative principle of the surgeons. It is a controversial whether residual carcinoma in situ (CIS) is truly an adverse prognostic factor [7–9]. More recently, there were several reports which suggested that R1-CIS increased the incidence of local recurrence and shortened postoperative survival against R0 resection [11–13].

Nevertheless, R1 resection has been consistently considered as an adverse prognostic factor and use of adjuvant chemoradiation to sterilize the microscopic residual diseases has been strongly recommended [11–14]. Unfortunately, for R1 disease, local failure rates are still unsatisfactory even after adjuvant chemoradiation employing conventional dose of 40-50 Gy [15–17]. There seems to be a room for improvement with respect to the local control. In definitive settings, radiation dose escalation demonstrated enhanced local control and overall survival (OS) in several previous studies for biliary tract cancers [18–21]. Therefore, a similar strategy could also be considered for patients with postoperative microscopic residuum. But to date, there is a paucity of information regarding optimal radiation dose after R1 resection in patients with EHBD cancer. This study was conducted to evaluate the impact of radiotherapy (RT) dose in patients with EHBD cancer after R1 resection.

## RESULTS

### Patient characteristics

Overall, there were 54 (62.8%) men and 32 (37.2%) women with a median age of 63 years (range, 38-86). Eastern cooperative oncology group (ECOG) performance status was mainly 0-1 in 74 (86.0%) patients. Proportion of older patients, defined as older than 60 years of age, was significantly higher in escalated dose group (53.4% vs 78.4%, *p* = 0.024). Although not statistically significant, more patients in escalated dose group had poor performance (ECOG 2-3, 9.3% vs 21.9%, *p* = 0.119). Distribution of other characteristics, such as primary tumor location, margin pathology, T stage, N stage, and tumor differentiation, was not significantly different between the two groups (Table 1).

**Table 1:** Comparison of characteristics between patients receiving conventional dose and those receiving escalated dose

Characteristics		Conventional dose group (*n*=54)	Escalated dose group (*n*=32)	*P*-value
Age (years)	≥60	29 (53.7%)	25 (78.1%)	0.024
	<60	25 (46.3%)	7 (21.9%)	
Gender	Male	32 (59.3%)	22 (68.8%)	0.379
	Female	22 (40.7%)	10 (31.2%)	
Performance status (ECOG)	0-1	49 (90.7%)	25 (78.1%)	0.119
	2-3	5 (9.3%)	7 (21.9%)	
Tumor location	Hilar	28 (51.9%)	18 (56.3%)	0.474
	Non-hilar	24 (44.4%)	11 (34.4%)	
	Diffuse	2 (3.7%)	3 (9.4%)	
Type of surgery	Bile duct resection	17 (31.5%)	10 (31.3%)	0.626
	Hepatobiliary resection	17 (31.5%)	11 (34.4%)	
	Pancreaticoduodenectomy	20 (37.0%)	11 (34.4%)	
Margin pathology	Invasive carcinoma	42 (77.8%)	21 (65.6%)	0.218
	Carcinoma in situ	12 (22.2%)	11 (34.4%)	
Histologic differentiation	WD, MD	47 (87.0%)	28 (87.5%)	0.670
	PD	3 (5.6%)	3 (9.4%)	
	Unknown	4 (7.4%)	1 (3.1%)	
Pathologic T stage	T1-2	30 (55.6%)	18 (56.3%)	0.950
	T3-4	24 (44.4%)	14 (43.8%)	
Pathologic N stage	N0	32 (59.3%)	21 (65.6%)	0.814
	N1	15 (27.8%)	8 (25.0%)	
	Nx	7 (13.0%)	3 (9.4%)	
Maintenance chemotherapy	Yes	36 (66.7%)	22 (68.8%)	0.842
	No	18 (33.3%)	10 (31.2%)	
Preoperative CA19-9	≥37 U/ml	26 (48.1%)	18 (56.3%)	0.989
	<37 U/ml	16 (29.6%)	11 (34.4%)	
	Unknown	12 (22.2%)	3 (9.4%)	

Note: P values were calculated using chi-square test. Abbreviations: ECOG = eastern cooperative oncology group; WD = well-differentiated; MD = moderate-differentiated; PD = poor-differentiated.

### Patterns of failure

The median follow-up duration was 27 months for all patients and 107 months for survivors. The median follow-up duration for conventional dose group and escalated dose group was 26 months (range, 4-236, 149 for survivors) and 37 months (range, 7-110, 93 for survivors), respectively. The mean follow-up duration was 59 months for conventional dose group compared to 49 months for escalated dose group (*p* = 0.339).

Overall, disease recurrences were observed in 56 (65.1%) patients. First site of relapse for conventional dose group and escalated dose group was as follows, respectively: locoregional recurrence (LRR) in 14 (25.9%) patients and 5 (15.6%) patients, DM in 15 (27.8%) patients and 13 (40.6%) patients, simultaneous LRR and DM in 8 (14.8%) patients and 1 (3.1%) patient.

### Prognostic factors for locoregional control (LRC)

**Figure 1:**
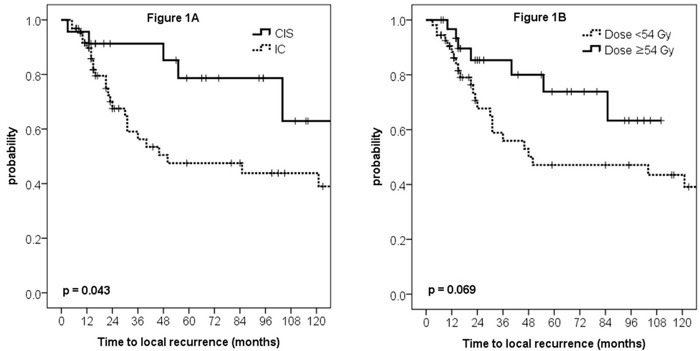
Locoregional control curves according to the margin pathology (**A**) and radiation dose (**B**).

The results of univariate analysis for LRC are shown in Table 2. LRC for patients with invasive carcinoma (IC) at RM vs. CIS at RM was 47.5% vs. 78.7% at 5 years, respectively (*p* = 0.043, Figure 1A). Escalated radiation dose showed marginally improved LRC (5yr rate, 73.8% vs. 47.1%, *p* = 0.069, Figure 1B). In multivariate analysis, factors with univariate p-value less than 0.2, which were margin pathology, RT dose, and preoperative CA 19-9, were incorporated into Cox proportional hazard model. As a result, IC at the margin (HR 2.957, 95% CI 1.096-7.976, *p* = 0.032) and radiation dose escalation (HR 0.394, 95% CI 0.158-0.986, *p* = 0.047) were independent prognostic factors for LRC (Table 2).

**Table 2:** Univariate and multivariate analyses for locoregional control

Variables	No.	5yr LRC	Univariate *P*	Multivariate* P	Hazard Ratio	95% CI
Age						
≥60	54	55.5%	0.562			
<60	32	59.8%				
Gender						
Male	54	57.9%	0.356			
Female	32	54.5%				
Tumor location						
Hilar	46	56.8%	0.851			
Non-hilar	35	57.9%				
Margin pathology						
Invasive carcinoma	63	47.5%	0.043	0.032	2.957	1.096-7.976
Carcinoma in situ	23	78.7%				
Histologic differentiation						
WD, MD	75	54.9%	0.677			
PD	6	66.7%				
Pathologic T stage						
T1-2	48	63.5%	0.345			
T3-4	38	52.7%				
Pathologic N stage						
N0	53	60.5%	0.500			
N1	23	43.3%				
Radiotherapy course						
Split	45	50.4%	0.272			
Continuous	41	64.3%				
Radiotherapy dose						
≥54 Gy	32	73.8%	0.069	0.047	0.394	0.158-0.986
<54 Gy	54	47.1%				
Maintenance chemotherapy						
Yes	58	54.8%	0.978			
No	28	61.9%				
Preoperative CA19-9						
≥37 U/ml	44	42.6%	0.145	0.147	1.865	0.803-4.333
<37 U/ml	27	69.1%				

Abbreviations: LRC = locoregional control; CI = confidence interval; WD = well-differentiated; MD = moderate-differentiated; PD = poor-differentiated.

* Factors with univariate p-value less than 0.2 were selected for Cox proportional hazard model.

However, evaluation of radiation dose effect on LRC in each subgroup of patients divided by RM status showed no statistical significances according to the dose group, despite numerical difference. In patients with IC (*n* = 63), 5yr LRC was 65.6% with escalated dose and 40.7% with conventional dose (*p* = 0.345, Supplementary Figure 1A). In patients with CIS (*n* = 23), 5yr LRC was 87.5% with escalated dose and 69.4% with conventional dose (*p* = 0.191, Supplementary Figure 1B).

### Impact of RT dose on survival outcomes

**Figure 2:**
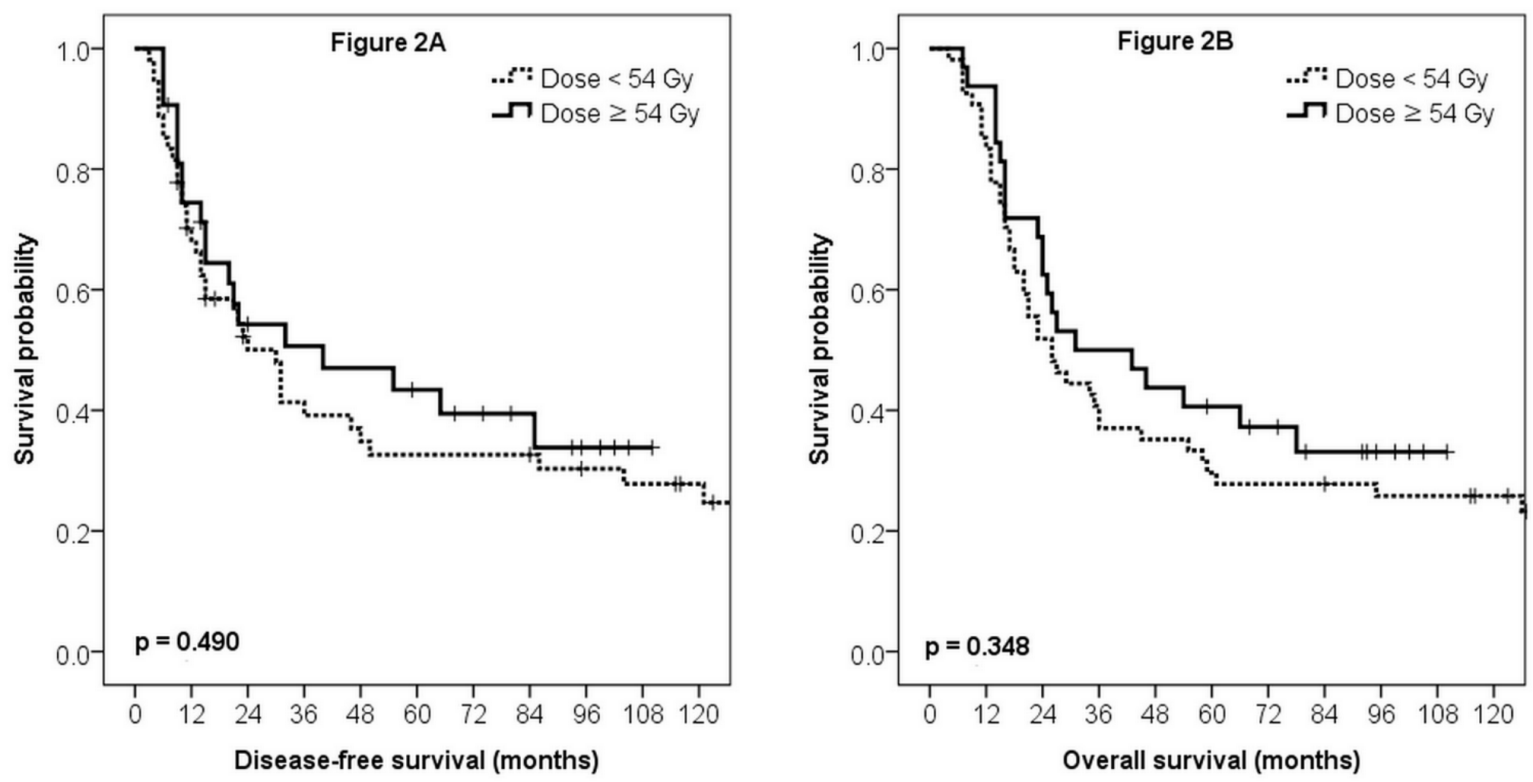
Disease-free survival (**A**) and overall survival (**B**) curves according to the radiation dose.

As for disease-free survival (DFS), multivariate analysis revealed that preoperative CA 19-9 value was the only factor significantly associated with DFS (*p* = 0.040, Supplementary Table 1), whereas pathologic N stage showed marginal significance (*p* = 0.063). Radiation dose was not significantly related with DFS (5yr rate, 43.4% vs. 32.6%, Figure 2A, *p* = 0.490).

As for OS, multivariate analysis revealed margin pathology as the sole significant prognosticator (*p* = 0.021, Supplementary Table 2). Preoperative CA 19-9 value showed marginal significance (*p* = 0.053), whereas, radiation dose did not show statistical significance (5yr rate, 40.6% vs. 29.6%, Figure 2B, *p* = 0.348).

**Figure 3:**
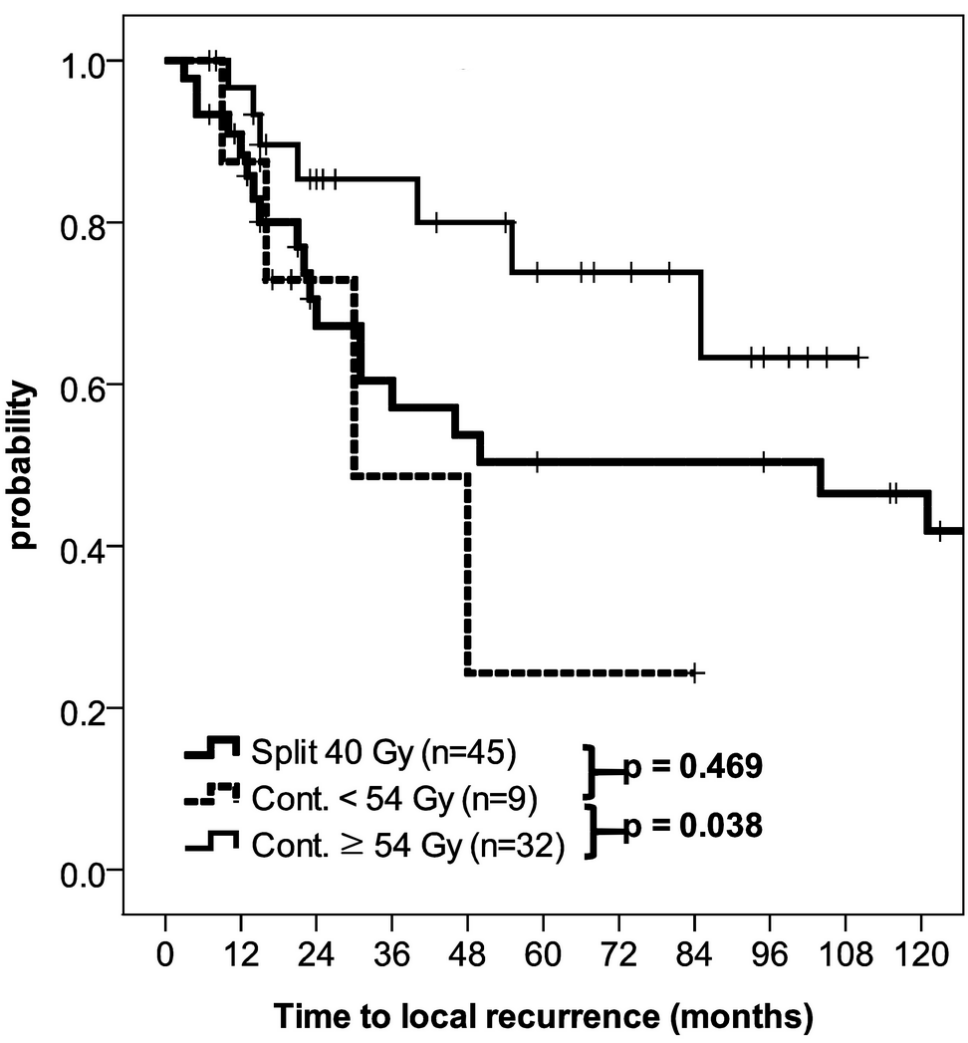
Locoregional control curves according to the radiation course (split vs. continuous) and dose

Because split course RT may have had an adverse impact on LRC, further subgroup analysis was performed. In conventional dose group, 9 and 45 patients underwent continuous course and split course, respectively. There was no significant difference between LRC of patients with split course RT against those with continuous course (Figure 3, *p* = 0.469). Conversely, for the patients undergoing continuous course RT, LRC difference was significant for radiation dose; 5yr LRC estimates for ≥ 54 Gy vs. ≤ 50.4 Gy were 73.8% vs. 24.3%, respectively (Figure 3, *p* = 0.038).

### Acute and late toxicities

Between the two dose groups, there was no significant difference in acute gastrointestinal (GI) toxicity ≥ grade 2 and in late GI toxicity ≥ grade 3 (Table 3). Acute GI toxicities were mostly less than grade 3, which were tolerable and alleviated after supportive treatments. About 6% of patients had late GI complications of grade 3 or greater in both groups (p > 0.99). During chemoradiation, hematologic toxicities were relatively mild and there was no significant difference between two groups (Table 3).

**Table 3:** Acute and late toxicities

	Conventional dose group (*n*=54)	Escalated dose group (*n*=32)	*P*-value
Acute gastrointestinal ≥ grade 2	30 (56%)	12 (38%)	0.105
Late gastrointestinal ≥ grade 3	3 (6%)	2 (6%)	1.000
Hematologic ≥ grade 2	13 (24%)	13 (41%)	0.106
Hematologic ≥ grade 3	1 (2%)	2 (6%)	0.553

Note: P values were calculated using Fisher's exact test.

## DISCUSSION

Microscopic margin involvement after curative resection for EHBD cancer is not uncommon in current practice. However, the definitive role and optimal dose of RT are still unknown. To evaluate the impact of adjuvant radiation dose for R1 resection, we only included patients who underwent adjuvant RT for EHBD cancer. The results of the present study suggested that dose escalation to 54 Gy or higher resulted in significant improvement of LRC in patients after R1 resection.

To our knowledge, there has been no study evaluating radiation dose escalation after R1 resection in EHBD cancer. Im et al. reported that a postoperative RT dose < 50 Gy was suboptimal for OS and LRC in patients with EHBD cancer who had undergone curative resection [22]. However, they included patients with R0, R1 and R2 RM altogether, therefore impact of higher RT dose solely for R1 patients could not be assessed. Nevertheless, radiation dose ≥ 50 Gy was a significant prognostic factor for OS, event-free survival, and LRC in their cohort. Together with results from current analysis, radiation dose beyond 50 Gy may be required to improve LRC, irrespective of margin status.

Another important consideration other than nominal dose is a RT scheme. In current analysis, many patients in conventional dose group received 40 Gy-split course RT, which could be regarded as suboptimal in current standard, whereas all patient in escalated dose group underwent continuous treatment. There was significant LRC differences according to RT dose in the continuous group as shown in Figure 3. On the other hand, there was no LRC difference when split course and continuous course in the conventional dose group was compared. However, this may be due to small number of patients included for analysis. Furthermore, it should be noted that all patients included in current analysis had resection margin involvement. This increased tumor burden may be one of the underlying causes. There are conflicting reports on impact of RT scheme on local control and survival, possibly biased by nature of patient cohort [23–25]. Thus, findings from current studied should be interpreted with caution.

Although there is no prospective data demonstrating the influence of RT dose escalation, few retrospective studies have reported positive impact of RT dose escalation in the definitive setting. In the study by Alden et al., a dose response was suggested by increased median OS with RT dose escalation [19]. They suggested that higher radiation dose (60-75 Gy) delivery by combining brachytherapy was well tolerated and appeared to be an effective modality for unresected EHBD cancer. Similarly, improved local control with higher RT dose was also found in the MD Anderson Cancer Center study [20]. However, median survival was not increased. Aforementioned studies suggested that positive dose response relationship might exist and conventional dose of less than 50 Gy might be inadequate for local control in EHBD cancer.

Most retrospective studies of adjuvant chemoRT in patients with EHBD cancer have reported 40-70% of 5yr LRC after conventional dose RT [10, 15–17, 22, 26]. 5yr LRC of 73.8% in current study is comparable to or somewhat better than the previous results, considering that all patients included in current analysis underwent R1 resection. This also indirectly supports the role of RT dose escalation in these patients. However, improvement in LRC was not readily translated to improvement in either DFS or OS. This in turn meant DM was the major pattern of failure due to implementation of active loco-regional treatment, as previously suggested [26, 27]. Therefore, more effective systemic control should be considered to decrease DM for these patients. Gemcitabine/cisplatin combination have shown promising results in the treatment of metastatic biliary tract cancer, and more recently, similarly in the adjuvant setting [28–30]. Thus, gemcitabine based adjuvant chemotherapy may improve treatment results over 5-FU based therapy as employed in current study. Moreover, optimal chemotherapeutic agents for combining radiation dose escalation need to be further studied.

However, RT dose escalation could increase treatment-related adverse event. In current study, median follow-up period for survivors were 149 months in conventional dose group and 93 months in escalated dose group, which may be long enough to detect long-term complications. Despite limitation of being a retrospective analysis, late radiation-related GI toxicity was not increased with RT dose escalation even after extended follow up. In this study, majority of toxicities were of low grade and thus well tolerated. One of key issue to lower manageable toxicity may be limited radiation volume. Most of the RT field received 45 Gy and only focal area with R1 RM received 54 Gy or higher dose with highly conformal technique. Similarly, intensity-modulated radiation therapy have shown improved dose distributions to dose-limiting normal organs near the target among patients with upper abdominal malignancies [31, 32]. Other techniques such as three-dimensional brachytherapy planning or stereotactic body RT is also advancing and improving. Therefore, dose escalation strategy could be more readily applied with up-to-date RT techniques, without increased toxicities.

However, there are several weaknesses in the present study. First and foremost, the limitations inherent in retrospective analysis are challenging to overcome. Further validation with external cohort or data analysis from prospective study is indeed mandatory to support the notions made through current analysis. Second, radiation-related toxicities, especially those of less than grade 3, may have been underestimated. However, grade 3 or higher GI toxicity typically warrants hospitalization and parenteral support, which would have been documented in our dataset. This may reduce, but does not completely eliminate, the possibility of underestimation of severe GI toxicity. Despite these shortcomings, strengths of the present study are the inclusion of a relatively homogenous group of high-risk patients and the sufficient follow-up period.

In conclusion, current study demonstrated that radiation dose ≥ 54 Gy was associated with improved LRC in patients after R1 resection and was also well tolerated. Therefore, dose escalation could be considered for patients with R1 resection. Improvement in LRC with radiation dose escalation coupled with improved systemic control may eventually contribute to improved DFS and OS. Further validation study is warranted.

## MATERIALS AND METHODS

### Study population

After institutional review board approval, we retrospectively searched eligible patients who received adjuvant RT after resection with curative intent between Jan 1995 to Dec 2009. Patients with gross residual disease after resection were initially excluded during the search process together with patients who underwent palliative resection. Among 251 patients after initial screening, 87 patients underwent R1 resection. One patient who refused treatment after 34 Gy was excluded in further analysis. R1 resection was defined as the presence of IC (*n* = 63) or CIS (*n* = 23) at any side of resected specimen, which were confirmed in permanent pathologic reports. High-grade dysplasia was grouped as CIS due to extreme difficulty of distinguishing two epithelial lesions.

### Surgery and staging

The types and extents of surgery were primarily determined by surgeon taking into account of various aspects including the location of primary tumor, frozen pathologic section reviews during surgery, and risk of postoperative morbidity. Pancreaticoduodenectomy was performed in 31 patients, bile duct resection was performed in 27 patients, and bile duct resections with varying extent of partial hepatectomy was performed in 28 patients. The detailed surgical principles and techniques used have been previously described [12].

For staging purposes, American Joint Committee on Cancer (AJCC) staging 6th edition was used in order to apply same staging system to all studied patients independent of primary tumor location.

### Adjuvant treatment

The detailed RT techniques for EHBD cancer have also been previously described [27]. All patients’ RT planning were individualized and conducted by treatment planning software. In 45 patients treated till early 2000’s, radiation dose of 40 Gy in 20 daily fractions was delivered to tumor bed and regional lymph nodes, with 2 weeks of planned rest after 20 Gy following the GITSG trial protocol [33]. Concomitant 5-fluorouracil (5-FU, 500mg/m^2^/day intravenous bolus) was administered for the first 3 days of each 2 weeks of RT. Remaining 41 patients underwent continuous course RT. Nine patients received 50 or 50.4 Gy and 32 patients received additional boost to tumor bed or R1 RM upto 54-56 Gy. A median dose of 45 Gy was delivered to the regional nodal area and a cone-down volume including only tumor bed with R1 margin received an additional boost. All 32 patients with radiation dose ≥ 54 Gy underwent continuous course RT. Concomitant fluoropyrimidine-based chemotherapy (intravenous 5-FU or oral capecitabine) was given during continuous course of RT. Maintenance chemotherapy was also administered to 58 (67.4%) patients after the completion of RT.

### Toxicity evaluation

Acute and late GI toxicity were evaluated using Radiation Therapy Oncology Group criteria [34]. Briefly, acute GI toxicity grade 2 refers to anorexia with ≤ 15% weight loss from pretreatment baseline, nausea and/or vomiting requiring antiemetics, abdominal pain requiring analgesics or diarrhea requiring parasympatholytic drugs. Late GI toxicity grade 3 is defined as obstruction or bleeding, requiring surgery. However, RTOG toxicity criteria does not have separate category for bile duct tissue, but small/large intestine categories, thus were counted accordingly. Therefore, biliary stricture causing recurrent cholangitis was regarded as an obstruction, which is grade 3 in small/large intestinal toxicity, and were counted as such. During chemoRT, hematologic toxicities were assessed by Common Terminology Criteria for Adverse Events 4.0.

### Statistical analysis

We divided patients into two groups in later analysis: conventional dose group (radiation dose 40-50.4 Gy) vs. escalated dose group (radiation dose 54-56 Gy). Primary endpoint was a LRC rate. LRR was detected by imaging study (computed tomography or positron-emission tomography), which was defined as the tumor recurrence in the surgical bed or regional nodal area. As secondary endpoints, DFS was defined as the time interval between the date of surgery and any failure or death during follow-up period. Overall survival was also measured from the date of surgery to the date of death from any cause or the last follow-up. The actuarial survival rates were calculated by the Kaplan–Meier method and compared using the log-rank test. The Cox proportional hazards model was applied for multivariate analysis. The differences of clinical and tumor characteristics between two dose groups were compared using Fisher exact test or chi-square test. Data were analyzed using SPSS software, release 18.0.1 (SPSS Inc. Chicago, IL, USA).

## SUPPLEMENTARY MATERIALS FIGURE AND TABLE




