# Supplementary material for: Impact of radiation dose in postoperative radiotherapy after R1 resection for extrahepatic bile duct cancer: long term results from a single institution
Source: Oncotarget. 2017 Apr 21;8(44):78076–85. doi: 10.18632/oncotarget.17368 (PMC5652838; doi:10.18632/oncotarget.17368)
Supplement: Supplementary file 1 [file oncotarget-08-78076-s001.pdf]

Impact of radiation dose in postoperative radiotherapy after R1 resection for extrahepatic bile duct cancer: long term results from a single institution

SUPPLEMENTARY MATERIALS

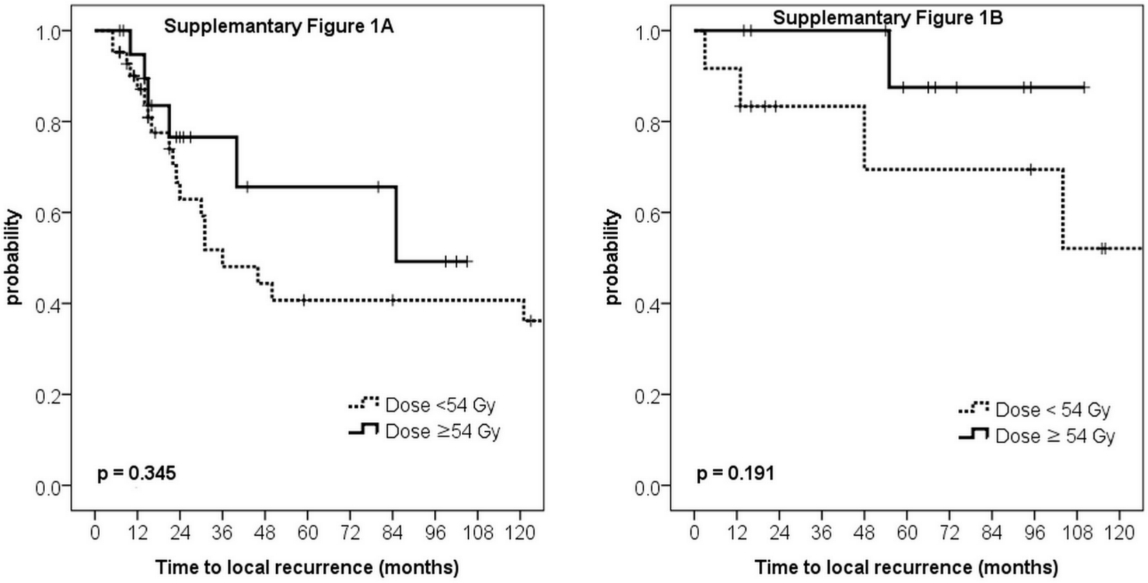

Supplementary Figure 1: Locoregional control curves according to the radiation dose in patients with invasive carcinoma at the margin (**A**) and carcinoma in situ at the margin (**B**).

For Supplementary Tables 1 & 2 see in Supplementary Files.
