# Supplementary material for: Impact of radiation dose in postoperative radiotherapy after R1 resection for extrahepatic bile duct cancer: long term results from a single institution
Source: Oncotarget. 2017 Apr 21;8(44):78076–85. doi: 10.18632/oncotarget.17368 (PMC5652838; doi:10.18632/oncotarget.17368)
Supplement: Supplementary file 2 [file oncotarget-08-78076-s002.docx]

Supplementary Table 1: Univariate and multivariate analyses for disease-free survival

| Variables | No. | 5yr DFS | Univariate  P | Multivariate  P | Hazard Ratio | 95% CI |
| --- | --- | --- | --- | --- | --- | --- |
| Age |  |  |  |  |  |  |
| ≥60 | 54 | 36.7% | 0.941 |  |  |  |
| <60 | 32 | 36.9% |  |  |  |  |
| Gender |  |  |  |  |  |  |
| Male | 54 | 36.7% | 0.721 |  |  |  |
| Female | 32 | 36.3% |  |  |  |  |
| Tumor location |  |  |  |  |  |  |
| Hilar | 46 | 35.1% | 0.469 |  |  |  |
| Non-hilar | 35 | 38.6% |  |  |  |  |
| Margin pathology |  |  |  |  |  |  |
| Invasive carcinoma | 63 | 28.8% | 0.097 | 0.149 | 1 |  |
| Carcinoma in situ | 23 | 55.9% |  |  | 0.588 | 0.286-1.209 |
| Histologic differentiation |  |  |  |  |  |  |
| WD, MD | 75 | 36.6% | 0.197 |  |  |  |
| PD | 6 | 16.7% |  |  |  |  |
| Pathologic T stage |  |  |  |  |  |  |
| T1-2 | 48 | 42.7% | 0.074 | 0.364 | 1 |  |
| T3-4 | 38 | 29.5% |  |  | 1.336 | 0.715-2.498 |
| Pathologic N stage |  |  |  |  |  |  |
| N0 | 53 | 41.6% | 0.095 | 0.063 | 1 |  |
| N1 | 23 | 22.4% |  |  | 1.867 | 0.967-3.606 |
| Radiotherapy course |  |  |  |  |  |  |
| Split | 45 | 39.0% | 0.811 |  |  |  |
| Continuous | 41 | 34.6% |  |  |  |  |
| Radiotherapy dose |  |  |  |  |  |  |
| ≥54 Gy | 32 | 43.4% | 0.490 |  |  |  |
| <54 Gy | 54 | 32.6% |  |  |  |  |
| Maintenance chemotherapy |  |  |  |  |  |  |
| Yes | 58 | 34.6% | 0.812 |  |  |  |
| No | 28 | 42.4% |  |  |  |  |
| Preoperative CA19-9 |  |  |  |  |  |  |
| ≥37 U/ml | 44 | 23.3% | 0.012 | 0.040 | 2.125 | 1.035-4.360 |
| <37 U/ml | 27 | 58.9% |  |  | 1 |  |
| *Abbreviations:* DFS = disease-free survival; CI = confidence interval; WD = well-differentiated; MD = moderate-differentiated; PD = poor-differentiated. | | | | | | |

Supplementary Table 2: Univariate and multivariate analyses for overall survival

| Variables | No. | 5yr OS | Univariate  P | Multivariate  P | Hazard Ratio | 95% CI |
| --- | --- | --- | --- | --- | --- | --- |
| Age |  |  |  |  |  |  |
| ≥60 | 54 | 33.3% | 0.638 |  |  |  |
| <60 | 32 | 34.4% |  |  |  |  |
| Gender |  |  |  |  |  |  |
| Male | 54 | 33.3% | 0.390 |  |  |  |
| Female | 32 | 34.4% |  |  |  |  |
| Tumor location |  |  |  |  |  |  |
| Hilar | 46 | 28.3% | 0.332 |  |  |  |
| Non-hilar | 35 | 37.1% |  |  |  |  |
| Margin pathology |  |  |  |  |  |  |
| Invasive carcinoma | 63 | 25.4% | 0.043 | 0.021 | 1 |  |
| Carcinoma in situ | 23 | 56.5% |  |  | 0.463 | 0.241-0.891 |
| Histologic differentiation |  |  |  |  |  |  |
| WD, MD | 75 | 33.3% | 0.282 |  |  |  |
| PD | 6 | 16.7% |  |  |  |  |
| Pathologic T stage |  |  |  |  |  |  |
| T1-2 | 48 | 39.6% | 0.135 |  |  |  |
| T3-4 | 38 | 26.3% |  |  |  |  |
| Pathologic N stage |  |  |  |  |  |  |
| N0 | 53 | 37.7% | 0.122 |  |  |  |
| N1 | 23 | 21.7% |  |  |  |  |
| Radiotherapy course |  |  |  |  |  |  |
| Split | 45 | 33.3% | 0.943 |  |  |  |
| Continuous | 41 | 34.1% |  |  |  |  |
| Radiotherapy dose |  |  |  |  |  |  |
| ≥54 Gy | 32 | 40.6% | 0.348 |  |  |  |
| <54 Gy | 54 | 29.6% |  |  |  |  |
| Maintenance chemotherapy |  |  |  |  |  |  |
| Yes | 58 | 32.8% | 0.953 |  |  |  |
| No | 28 | 35.7% |  |  |  |  |
| Preoperative CA19-9 |  |  |  |  |  |  |
| ≥37 U/ml | 44 | 22.7% | 0.048 | 0.053 | 1.799 | 0.993-3.259 |
| <37 U/ml | 27 | 51.9% |  |  | 1 |  |
| *Abbreviations:* OS = overall survival; CI = confidence interval; WD = well-differentiated; MD = moderate-differentiated; PD = poor-differentiated. | | | | | | |
